# Zinc Oxide Nanoparticle Synergizes Sorafenib Anticancer Efficacy with Minimizing Its Cytotoxicity

**DOI:** 10.1155/2020/1362104

**Published:** 2020-05-28

**Authors:** Ahmed Nabil, Mohamed M. Elshemy, Medhat Asem, Marwa Abdel-Motaal, Heba F. Gomaa, Faten Zahran, Koichiro Uto, Mitsuhiro Ebara

**Affiliations:** ^1^Research Center for Functional Materials, National Institute for Materials Science (NIMS), 1-1 Namiki, Tsukuba, Ibaraki 305-0044, Japan; ^2^Biotechnology and Life Sciences Department, Faculty of Postgraduate Studies for Advanced Sciences (PSAS), Beni-Suef University, Beni-Suef, Egypt; ^3^Faculty of Science, Menoufia University, Menoufia, Egypt; ^4^Department of Chemistry, Faculty of Science, Mansoura University, Mansoura, Egypt; ^5^Chemistry Department, College of Science, Qassim University, Qassim, Saudi Arabia; ^6^Zoology Department, Faculty of Science, Ain-Shams University, Biology Department, Faculty of Sciences and Arts-Scientific Departments, Qassim University, Saudi Arabia; ^7^Biochemistry Department, Faculty of Science, Zagazig University, Egypt; ^8^Graduate School of Pure and Applied Sciences, University of Tsukuba, 1-1-1 Tennodai, Tsukuba, Ibaraki 305-8577, Japan; ^9^Graduate School of Industrial Science and Technology, Tokyo University of Science, 6-3-1 Niijuku, Katsushika-ku, Tokyo 125-8585, Japan

## Abstract

Cancer, as a group, represents the most important cause of death worldwide. Unfortunately, the available therapeutic approaches of cancer including surgery, chemotherapy, radiotherapy, and immunotherapy are unsatisfactory and represent a great challenge as many patients have cancer recurrence and severe side effects. Methotrexate (MTX) is a well-established (antineoplastic or cytotoxic) chemotherapy and immunosuppressant drug used to treat different types of cancer, but its usage requires high doses causing severe side effects. Therefore, we need a novel drug with high antitumor efficacy in addition to safety. The aim of this study was the evaluation of the antitumor efficacy of zinc oxide nanoparticle (ZnO-NPs) and sorafenib alone or in combination on solid Ehrlich carcinoma (SEC) in mice. Sixty adult female Swiss-albino mice were divided equally into 6 groups as follows: control, SEC, MTX, ZnO-NPs, sorafenib, and ZnO-NPs+sorafenib; all treatments continued for 4 weeks. ZnO-NPs were characterized by TEM, zeta potential, and SEM mapping. Data showed that ZnO-NPs synergized with sorafenib as a combination therapy to execute more effective and safer anticancer activity compared to monotherapy as showed by a significant reduction (*P* < 0.001) in tumor weight, tumor cell viability, and cancer tissue glutathione amount as well as by significant increase (*P* < 0.001) in tumor growth inhibition rate, DNA fragmentation, reactive oxygen species generation, the release of cytochrome c, and expression of the apoptotic gene caspase-3 in the tumor tissues with minimal changes in the liver, renal, and hematological parameters. Therefore, we suggest that ZnO-NPs might be a safe candidate in combination with sorafenib as a more potent anticancer. The safety of this combined treatment may allow its use in clinical trials.

## 1. Introduction

Each year, tens of millions of people are diagnosed with cancer around the world. As concerns mortality, cancer is considered the second cause of death throughout the world and will soon become the first cause of death in many parts of the globe ([1, 2]. Unfortunately, the available therapeutic and diagnostic approaches of cancer are unsatisfactory and represent a great challenge as many patients have cancer recurrence and severe side effects [3]. So, there are increasing demands for investigation and identification of new drugs as antitumor therapy with low side effects [4].

SEC is an undifferentiated solid carcinoma derived from mammary adenocarcinoma in mice (Sakai et al., 2010) which has a high transplantable capacity, rapidly growing tumor, short life span, and 100% malignancy [5] and is used as an experimental model to investigate the anticancer activity of drugs or natural compounds [6].

Chemotherapy is one of the most common and effective treatments for cancer which kills tumor cells using genotoxicity. However, it also harms normal cells that cause diverse dose-dependent side effects such as fatigue, loss of appetite, nausea, bowel issues, hair loss, skin discoloration, and even death in extreme cases [7]. MTX is a chemotherapeutic agent that was firstly used in the treatment of solid cancers by (Pierce and Dixon, 1958). Also, it is used in the treatment of various types of tumors and autoimmune diseases [8] due to its ability to hinder cell proliferation and synthesis of nucleotide and proteins by suppression of dihydrofolate reductase of folate metabolic pathway that plays a key role in nucleotide biosynthesis pathway [9]. Moreover, MTX derivatives like pemetrexed suppress enzymes involved in purine and pyrimidine metabolism, impairing RNA and DNA synthesis in tumors [10]. Previous studies proposed that coassembly of hydroxycamptothecin and MTX followed by surface covering through acidity-responsive polyethylene glycol might be a promising strategy for synergistically enhancing chemotherapy efficiency with minimized side effect synergistic therapeutic function [11].

Tyrosine kinase inhibitors (TKIs) are a pharmaceutical drug including three generations (first, second, and third generation) that inhibits tyrosine kinase enzymes that compete with ATP for the ATP binding site of protein tyrosine kinase and reduce tyrosine kinase phosphorylation inhibiting tumor cell proliferation. Sorafenib, a systematic multikinase inhibitor with antiproliferative properties, has been used as the first-line drug for advanced hepatocellular carcinoma patients as it suppresses tumor cells' growth and proliferation by inhibition of serine/threonine kinase and other tyrosine kinase signalling pathways [12].

ZnO-NPs have received considerable attention in various fields due to their excellent physicochemical properties, safety, biodegradability [13], and their fast delivery to different tissues and organs in addition to various biological purposes including drug delivery and immune-modulatory agent (Kalpana et al., 2018; [14]). ZnO-NPs have shown a promising anticancer behaviour besides its therapeutic activity against diabetes, microbial infections, inflammations, and wound healing [15]. Regarding cancer treatment, ZnO-NPs were approved to have a potential molecular effect including a reduction in cellular viability, loss of membrane integrity, and activation of the programmed cell death (apoptosis) [16]. It is now clear that ZnO-NPs possess a kind of cytotoxicity against tumor cells with a minimum injury to healthy cells [17]. Therefore, in the present study, we aimed to evaluate the anticarcinogenic potency of sorafenib and ZnO-NPs alone and in combination against solid Ehrlich carcinoma compared with FDA-approved chemotherapeutic agent MTX.

## 2. Materials and Methods

### 2.1. Drugs and Chemicals

MTX was obtained from Sandoz Limited, a Novartis division, UK. Sorafenib (formerly Nexavar®) was generously supplied by Bayer AG of Germany, while zinc acetate dihydrate, ethane-1, 2-diol, and triglycol were obtained from Sigma-Aldrich Chemical Co. (St. Louis, MO, USA). Other chemicals and reagents used were of the highest purity grade.

### 2.2. Induction of Solid Ehrlich Carcinoma (SEC) Tumor in Mice

A model of SEC used, for Ehrlich carcinoma cells (ECC), was obtained from the National Cancer Institute, Cairo University (Giza, Egypt). Mice were implanted subcutaneously with 2 × 10^6^ Ehrlich carcinoma cells into the right thigh of the hind limb [18]. A solid tumor mass developed within 12 days after implantation.

### 2.3. Animals and Experimental Design

This study was carried out on sixty adult female Swiss-albino mice weighting approximately 22-29 g, which were purchased from Medical Experimental Research Centre, Faculty of Medicine, Mansoura University (MERC), Mansoura, Egypt. Mice were kept in an air-conditioned animal house with specific pathogen-free conditions with a 12 : 12 h daylight/darkness and provided food and water ad libitum. All the procedures relating to animal care and treatments strictly adhered to the Guide for the Care and Use of Laboratory Animals published by the US National Institutes of Health (Publication No. 85-23, revised 1996). Mice were divided equally into 6 groups as follows:

*Group I*: 10 mice were injected with saline and kept as healthy control.

*Group II*: 10 mice were implanted subcutaneously with 2 × 10^6^ Ehrlich carcinoma cells into the right thigh of the hind limb, injected with saline instead of treatment, and kept as the untreated control.

*Group III*: 10 mice were implanted subcutaneously with 2 × 10^6^ Ehrlich carcinoma cells into the right thigh of the hind limb then treated with MTX (2.5 mg/kg/I.P.) every day [19].

*Group IV*: 10 mice were implanted subcutaneously with 2 × 10^6^ Ehrlich carcinoma cells into the right thigh of the hind limb then treated with 5 mg/kg of ZnO-NPs I.P. every day [20].

*Group V*: 10 mice were implanted subcutaneously with 2 × 10^6^ Ehrlich carcinoma cells into the right thigh of the hind limb then treated with 30 mg/kg sorafenib orally every day [21].

*Group VI*: 10 mice were implanted subcutaneously with 2 × 10^6^ Ehrlich carcinoma cells into the right thigh of the hind limb then treated with 5 mg/kg of ZnO-NPs I.P. plus 30 mg/kg sorafenib orally every day.

### 2.4. Collection and Preparation of Samples

At the end of the experiment (4 weeks), all animals were euthanized by decapitation, and blood samples collected for biochemical and tumor markers investigations. The tumor was excised, weighed, then homogenized/decellularized for cell viability assay, DNA content, flow cytometry apoptotic markers, oxidation assay, and tumor growth inhibition (% TGI) calculation.

### 2.5. Hematological and Biochemical Analysis

Blood samples were used for determining haemoglobin (Hb), red blood cells (RBCs), and leucocytes using the Sysmex XT 2000 Haematology Autoanalyzer (Sysmex, Kobe, Japan) according to the manufacturer's recommendation. The levels of aspartate aminotransferase (AST) and alanine aminotransferase (ALT) were measured as liver injury markers; urea and creatinine levels were measured as renal injury markers using assay kits supplied by Spinreact Diagnostics, Girona, Spain.

### 2.6. Synthesis and Characterization of the ZnO-NPs

ZnO-NPs were synthesized by refluxing its precursor zinc acetate dihydrate (0.1 M) in ethane-1,2-diol and triglycol at 180 and 220°C, respectively. The time of the reaction varied for 2 or 3 h in the presence and absence of sodium acetate (0.01 M). The solution was put on a magnetic stirrer at 80°C (1.5 h) then centrifuged at 8000 rpm (15 min) and rinsed with deionized water and ethyl alcohol 3 times. Finally, it was dried overnight at 80°C. ZnO-NP dose was dissolved in deionized water till the complete dissolution. Size, morphology, and elemental composition were observed and measured by a transmission and scanning electron microscope (TEM and SEM) (JEOL, Japan), while the surface zeta potential measurements were also measured by a zeta potential analyzer (Malvern Device, UK) [22].

### 2.7. Cell Viability Assay

Cell viability was determined in all groups using the 3-(4,5-dimethylthiazol-2-yl)-2,5-diphenyltetrazolium bromide (MTT) assay [23]. In brief, the solid tumor was decellularized; then, the cell suspension was seeded in 96-well plates (Greiner, Frickenhausen, Germany) at a density of 1 × 10^4^ cells/well, incubated for 24 h at 37°C (5% CO_2_). After incubation, 100 *μ*L/well of 0.5 mg/mL MTT was added to each well then incubated for 4 h, and the absorbance was measured at 570 nm using an enzyme-linked immunosorbent assay (ELISA) reader [24].

### 2.8. DNA Damage

The solid tumor was homogenized in chilled homogenization buffer (Ultra-Turrax, IKA T25, Germany) (pH 7.5) to obtain a tissue suspension; then, a centrifugation step was done with the generation of two fractions (corresponding to intact and fragmented DNA, respectively), precipitation of DNA, hydrolysis, and colorimetric quantitation upon staining with diphenylamine (DPA), which binds to deoxyribose. Optical density was measured at 600 nm with a multiwell spectrophotometer reader.

### 2.9. Caspase-3 and Cytochrome c Analysis

Caspase-3 used for evaluation of apoptosis induction in tumor cells using Cell-Event Caspase-3 detection reagent (5 *μ*M in PBS with 5% FBS) for 30 min at 37°C was purchased from Thermo Fisher Scientific (USA). Tumor cells (1 × 10^6^) from different groups were incubated with the primary anti-mouse antibodies for 1 h; then, the secondary antibodies FITC-conjugated goat-anti-rabbit antibodies were added for 30 min at 37°C and analyzed by flow cytometry (FACSCalibur, BD Biosciences) using CellQuest software. The activity of cytochrome c was measured in the homogenized tumor tissues using the Human ELISA kit (Abcam, Cambridge, UK). Specific cytochrome c antibodies were precoated onto 96-well plates incubated at room temperature. Washed with wash buffer, a streptavidin-HRP conjugate was added to each well and incubated at room temperature, and unbound conjugates were washed away and TMB was added and catalyzed by HRP to produce a yellow color. The density of yellow color was directly proportional to the concentration of cytochrome c.

### 2.10. Oxidative Stress Assessment

Tumor was homogenized using a Branson Sonifier (250, VWR Scientific, Danbury, CT) in potassium phosphate buffer (pH 6.5, 1 : 10) then centrifuged at 10, 000 × g at 4°C for 20 min for the determination of antioxidant enzymes, tissue-reduced glutathione (GSH) [25], tissue malondialdehyde (MDA) [26], and nitric oxide (NO) [27]. The level of reactive oxygen species (ROS) was measured in the homogenized tumor tissues depending on using the cell-permeant reagent 2′,7′-dichlorofluorescein diacetate (DCFDA), a fluorogenic dye that measures hydroxyl, peroxyl, and another ROS activity within the cell. After diffusion into the cell, DCFDA is deacetylated by cellular esterases to a nonfluorescent compound, which is later oxidized by ROS into 2′,7′-dichlorofluorescein (DCF). DCF is a highly fluorescent compound. Briefly, homogenized tissues were stained by adding 100 *μ*L of DCFDA to each well and incubated for 45 min at 37°C in the dark. Blank wells (with nonstained cells) were also used as a control. The fluorescence intensity was measured using an Infinite® 200 PRO plate reader at Ex/Em. = 488/525 nm, and the values were expressed as fluorescence intensity (FU)/protein content (mg).

Moreover, catalase (CAT) and superoxide dismutase (SOD) enzyme activity in the homogenate was assayed using colorimetric diagnostic kits (Biodiagnostic, Cairo, Egypt) according to the manufacturer's instructions. Serum total antioxidant capacity (TAC) was determined according to the methods reported by Koracevic et al. [28].

### 2.11. Statistical Analyses

Data were analyzed using SPSS software version 22 for Windows (IBM, Armonk, NY, USA). Descriptive statistics were calculated in the form of mean ± standard deviation (SD). ANOVA and Tukey's post hoc tests were used for comparison between groups. A level of *P* < 0.05 was defined as statistically significant.

## 3. Results

### 3.1. ZnO-NP Characterization

**Figure 1:**
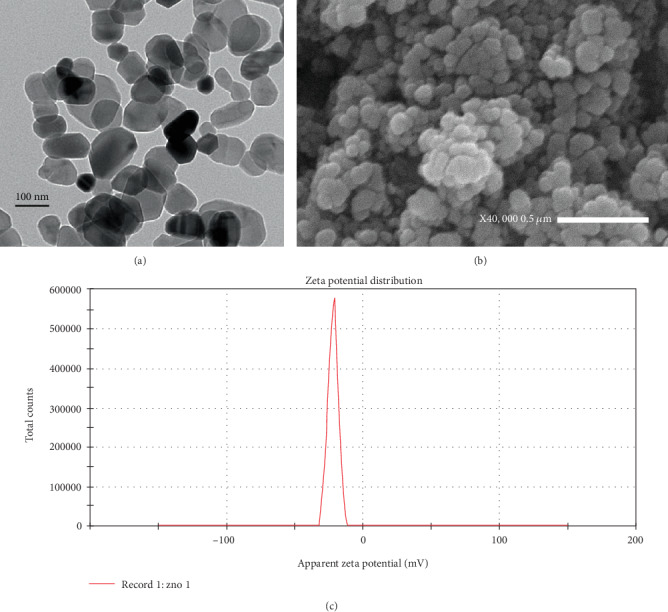
Characterization of ZnO-NPs: (a) TEM image of ZnO-NP average particle size = 37 nm; (b) SEM image of the synthesized ZnO-NPs; (c) zeta potential of ZnO-NPs.

ZnO-NP actual size and surface charge were confirmed by TEM, SEM, and a zeta potential analyzer. TEM micrographs revealed that ZnO-NPs were spheroid in shape (Figure 1(a)) and showed an average particle size = 37 nm; the zeta potential of ZnO-NPs was -22 mV, zeta deviation = 3.3 mV, and conductivity = 0.1 mS/cm (Figure 1(c)). SEM analysis of the synthesized ZnO-NPs shown in (Figure 1(b)) indicated the uniform grain appearance and the unique morphology without impurities.

### 3.2. Tumor Growth Inhibition Rate and the Difference in Tumor Weight

**Figure 2:**
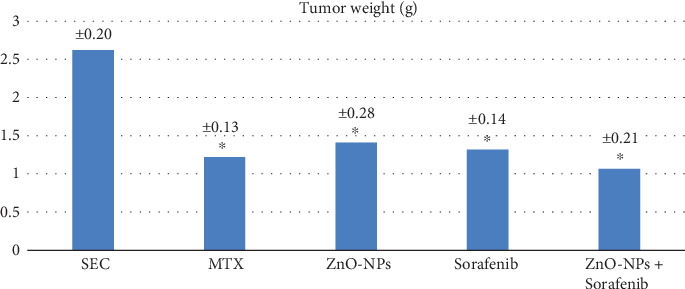
Effect of the treatment with MTX (2.5 mg/kg/I.P.), ZnO-NPs (5 mg/kg/I.P.), sorafenib (30 mg/kg/orally), and the combination of ZnO-NPs+sorafenib on SEC weight for 4 weeks. Results showed significant reductions (*P* < 0.001) in tumor weight in all treated groups compared to nontreated SEC group, and the best reduction in tumor (1.07 ± 0.21 g) weight was observed in the group that received combination therapy as ZnO-NPs synergized with sorafenib to execute the antitumor activity.

The administration of monotherapy MTX, ZnO-NPs, sorafenib, and ZnO-NPs+sorafenib combination showed significant reductions (*P* < 0.001) in tumor weight compared to the nontreated SEC group (G2). Moreover, G6 that received combination therapy (ZnO-NPs+sorafenib) showed the best reduction in tumor (1.07 ± 0.21 g) weight (Table 1, Figure 2). On the other hand, tumor growth inhibition % in different groups was 43.12%, 17.09%, 34.17%, and 47.23% for G3, G4, G5, and G6, respectively, which indicates that the combination treatment of ZnO-NPs+sorafenib significantly inhibits the tumor growth rate (*P* < 0.001) compared with the other monotherapy (Table 1).

### 3.3. Hematological and Biochemical Parameters

Table 2 showed a slight improvement in hematological parameters including Hb% and RBCs and significant improvement (*P* < 0.001) in leucocyte count towards normal observed with the different treatment regimens used in this experiment to antagonise the alterations induced by SEC in mice. Moreover, some liver and renal biomarker alterations induced by the tumor including AST and ALT as well as urea and creatinine significantly indicated liver and renal injury in the nontreated SEC group compared to the normal group. Conversely, different treatment regimens showed significant (*P* < 0.001) modulation in all liver and renal biochemical parameters while the combination therapy (ZnO-NPs+sorafenib) showed the most apparent improvement towards normal.

### 3.4. DNA Content

All treated groups showed a statistically significant decrease (*P* < 0.05) in DNA content compared with the untreated SEC group as shown in Table 3, especially with the combination therapy which decreases DNA content compared with other monotherapy.

### 3.5. Cell Viability Assay

Significant (*P* < 0.001) reduction in cell viability with different treatments compared to the SEC group was observed (Table 3). Also, the best cell viability reduction was observed with combined therapy.

### 3.6. Caspase-3 and Cytochrome c

**Figure 3:**
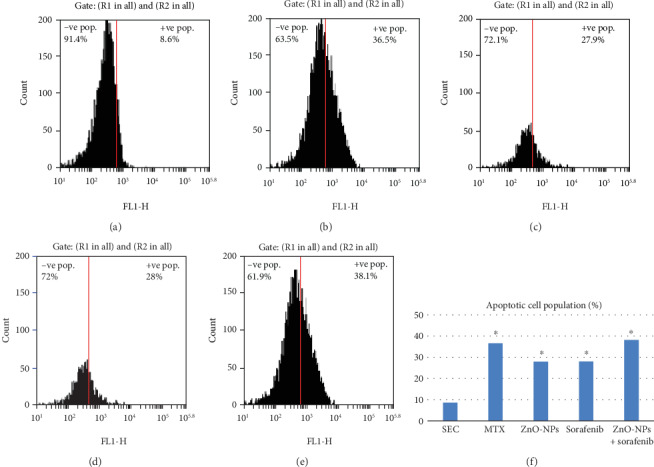
Caspase-3 flow cytometry analysis indicates apoptotic cell population % in all groups. (a) SEC group. (b) MTX group. (c) ZnO-NP group. (d) Sorafenib group. (e) ZnO-NPs+sorafenib group. (f) Apoptotic cell population (%).

As shown in Table 3 and Figure 3, MTX, ZnO-NPs, sorafenib, and the combination significantly release (*P* < 0.001) cytochrome c into the cytoplasm. Additionally, flow cytometry reported a marked increase in the expression of active caspase-3 with the different treatment regimens used in this experiment in comparison with the SEC group (*P* < 0.001). Furthermore, the combination therapy revealed a slight increase in the tissue expression of active caspase-3 and apoptotic cell population % compared to monotherapy (Figure 3(f)).

### 3.7. Oxidative Stress Assessment

The current study revealed a significant elevation in MDA level and ROS generation in the tumor tissues (*P* < 0.001) as well as a marked reduction in GSH content, CAT, and SOD enzyme activity in all treated groups compared to the nontreated SEC group tissues as shown in (Table 3). No significant difference was observed in the NO level between different study groups. Serum TAC of the SEC group significantly decreased as compared to that of all treated groups. Moreover, animals treated by ZnO-NPs combined with sorafenib reversed the antioxidant enzymes, MDA level, and serum TAC alterations towards the normal ranges compared with different monotherapy and SEC groups.

### 3.8. The Probable Anticancer Synergistic Mechanism between ZnO-NPs and Sorafenib

**Figure 4:**
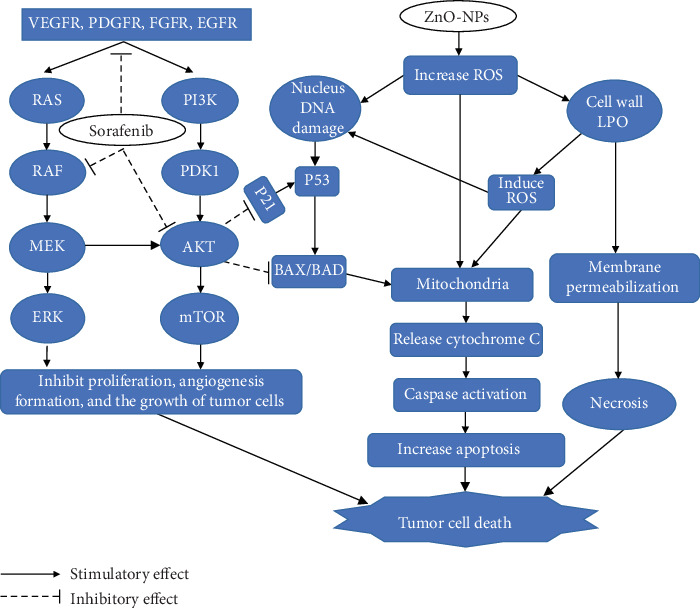
Schematic diagram of the probable mechanism of synergy between ZnO-NPs and sorafenib. Sorafenib has antagonist effect on vascular endothelial growth factor receptor (VEGFR-*β*), platelet-derived growth factor receptor (PDGFR), fibroblast growth factor receptor (FGFR), epidermal growth factor receptor (EGFR), protooncogene B-Raf, and protein kinase B-1 (AKT-1). However, ZnO-NPs are involved in reactive oxygen species (ROS) generation and increased cell wall lipid peroxidation (LPO) leading to cytotoxic and genotoxic effects. MEK: mitogen-activated protein kinase; ERK: extracellular signal-regulated kinase; PI3K: phosphatidylinositol-3-kinase; PDK1: pyruvate dehydrogenase lipoamide kinase isozyme 1; mTOR: mammalian target of rapamycin; p53: tumor protein p53; BAD: BCL-2-associated death promoter.

Figure 4 shows the possible mechanism by which ZnO-NPs synergized with sorafenib to execute a more effective and safer anticancer activity.

## 4. Discussion

Tumor treatment represents a challenging goal to find selective, effective, and safe therapy [29]. Ehrlich carcinoma has many advantages including the affordable cost, easily reproducible, and accessible to evaluate the efficacy and safety of anticancer therapies [30].

MTX is one of the most successful anticancer (antineoplastic or cytotoxic) chemotherapeutic drugs (used in high doses), but these doses had severe side effects. Therefore, we need new drugs with the same efficacy and high safety [31].

In this study, we evaluated the cytotoxic activity of sorafenib and ZnO-NPs alone and in combination against solid Ehrlich carcinoma compared with FDA-approved chemotherapeutic agent MTX.

Sorafenib, an oral multiple kinase inhibitor, significantly induces apoptosis in cancer model process, as well as inhibits tumor angiogenesis and cell proliferation to exert its anticancer activity, but it has severe cytotoxicity, leading to adverse events [32]. It was approved by the FDA as an effective therapy for advanced renal cell carcinoma in 2006 and advanced hepatocellular carcinoma in 2007 [33]. Being a multitarget kinase inhibitor, sorafenib blocks tyrosine kinase signalling receptors (VEGFR, PDGFR, and RET) and inhibits downstream Raf serine/threonine kinase activity to prevent tumor growth by antiangiogenic, antiproliferative, or proapoptotic effects that promote tumor cell apoptosis [34].

The previous studies found that ZnO-NPs have the potential to be used as anticancer therapy by targeting cancerous cells, enhancing cytotoxicity and cell death, which could be used as a foundation for developing new antitumor therapies [35]. ZnO-NPs did not show any kind of cytotoxicity in the liver and renal tissues when used as an anticancer agent [36].

It is generally admitted that positively charged nanoparticles have more affinity to be engulfed by cells than neutral or negative nanoparticles. It is supposedly due to favourable electrostatic interactions with the negatively charged cell membrane [37].

ZnO-NP surface has neutral hydroxyl groups which play an important role in the NP charging behaviour [38]. In alkaline pH, protons moved away from the metal surface inducing a negatively charged surface partly bonded oxygen atom (ZnO^−^); in acidic pH, H^+^ from the environment are likely moved to the NP surface, causing a positively charged surface (ZnOH_2_^+^). Under physiological state (acidic pH of tumor cells), the isoelectric point from 9 to10 shows that ZnO-NPs will possess a strongly positive-charged surface [39]. On the other hand, cancer cell outer layer membranes are characterized by the presence of a large number of anionic phospholipids [40]. Therefore, ZnO-NPs may be electrostatically attracted to the tumor tissues increasing cellular uptake of the nanoparticles [35]. In contrast, the normal healthy cells are either charge-neutral or slightly positive which show insignificant binding to the NPs [41].

Another important feature is that nanoparticles with size ≤ 100 nm remain in the circulation for a longer time and are able to avoid clearance by the reticuloendothelial system, also increasing intratumor concentrations [42]. Our data showed that the prepared ZnO-NP particle size average is equal to 37 nm and the zeta potential of ZnO-NPs was -22 mV; this stability and small particle size uniformity revealed the ability to target cancerous cells, enhancing cytotoxicity and cell death.

Our results are parallel to Xia et al. [43] who reported that ZnO-NPs induce ROS generation, enhancing cancer cytotoxicity and cell death. In agreement with other studies, different used treatment regimens especially the combination therapy significantly increase ROS levels which leads to oxidative damage to cellular structures and decreases antioxidant enzymes SOD and CAT activity in tumor tissue compared with the SEC group [44] as SOD catalyzes the dismutation of superoxide anion (O_2_^−^) to H_2_O_2_ and O_2_ [45]; also, CAT enzyme reduces H_2_O_2_ to H_2_O [43]. Treatment with ZnO-NPs showed a marked increase in MDA level which indicates lipid peroxidation and depletion of GSH in ESC tissues; these findings are in agreement with El-Shorbagy et al. [46].

Measuring the serum TAC may help to identify conditions affecting the oxidative status and the evaluation of physiological factors. TAC considers the cumulative action of all the antioxidants present in serum and body fluids, thus giving an insight into the delicate balance in vivo between oxidants and antioxidants [47]. Our data showed a significant reduction of serum TAC in the SEC and sorafenib groups due to oxidative stress and which is returning nearly to the basic state with MTX, ZnO-NPs, and the combined therapy (ZnO-NPs+sorafenib) with the best result observed with the combination. Previous parallel studies found that serum TAC decreased insignificantly in all groups when the concentration of zinc oxide nanoparticles was increased up to 200 mg/kg compared to the control group [48]. Another study [49] reported that there was no statistically significant alteration in serum levels of TAC, after 8 weeks of MTX dose up to 7.5 mg/kg therapy. Furthermore, Eisa et al. [50] reported that serum TAC of the Ehrlich carcinoma group significantly decreased as compared to that of the normal control group.

Consistent with our results, Diab et al. [51] revealed that oral administration of sorafenib in a dose of 10 mg/kg.b.wt. of rats daily for 2 weeks causes a significant decrease (*P* < 0.05) in total antioxidant capacity compared to the control healthy group. Moreover, Coriat et al. [52] reported that the sera of sorafenib-treated hepatocellular carcinoma patients contain increased levels of advanced oxidation protein products as sorafenib inhibits the MEK/ERK pathway that controls ROS production which exerts cytotoxic effects. Conversely, in animals treated by ZnO-NPs combined with sorafenib, the serum TAC level returning nearly to the basic state indicated that the combination had a less cytotoxic effect.

ROS including H_2_O_2_, ^·^OH, and ^·^O_2_^−^ may react with nucleophilic centres leading to DNA fragmentation and apoptosis upregulation, ultimately leading to carcinoma cell death [53]. Our findings agree with Bai et al. [54] who reported a significant increase in the DNA damage in the ZnO-NP-treated group compared with the nontreated SEC group.

The combined therapy of two or more therapeutic agents is a cornerstone of cancer treatment as it increases efficacy compared with monotherapy and may decrease the toxic effects on normal cells [55]. To the best of our knowledge, the current study was the first to evaluate the antitumor activity of sorafenib and ZnO-NP combined treatment. Our results showed that the tumor weight and growth inhibition rate significantly decreased with the different monotherapy treatment regimens compared to the nontreated SEC group as reported in previous studies [19, 56]; moreover, the best significant reduction in tumor weight (1.07 ± 0.21 g) and growth inhibition rate (47.23%) was observed in the combined therapy (ZnO-NPs+sorafenib) compared to monotherapy which demonstrates that the combined therapy had the best antitumor activity against SEC.

In the present study, Ehrlich tumor causes alterations in hematological parameters (Hb%, RBCs, and leucocytes) mainly due to iron deficiency as reported in previous studies [57]. Furthermore, our results were consistent with Borentain et al. [58] and Mutar et al. [59] who reported that SEC has led to liver and renal injury and marked changes in the liver and renal function in mice through an elevation in the levels of ALT, AST, urea, and creatinine. Conversely, animals treated by ZnO-NPs combined with sorafenib reversed the hematological and biochemical parameter alterations towards the normal ranges compared with different monotherapy and SEC groups which indicates a protective action on the liver, kidney, and the hematopoietic system against tumor cells in SEC mice.

The antiproliferative effect was determined using the MTT assay, which links directly to the mitochondrial enzymes [60]. Our results showed a significant reduction in cancer cell viability in the treated groups compared with the nontreated SEC group. Moreover, the highest reduction in cell viability was observed in the group treated with the combined therapy compared to monotherapy as ZnO-NPs synergized with sorafenib to execute the best antitumor activity.

ZnO-NPs induce oxidative damage of the plasma membrane which causes the loss of mitochondrial membrane potential, increased intracellular Ca_2_+ level, and release of cytochrome c which leads to the activation of caspases that enter the mitochondrial matrix to cleave key substrates in the electron transport chain [61]. Furthermore, sorafenib initiated apoptosis by cleavage of caspases and the mitochondrial release of cytochrome c [62]. Our data showed a marked increase in cytochrome c level and caspase-3 in the SEC tissues with both monotherapy and combination compared with the nontreated SEC group with the highest expression in the combined therapy group, as ZnO-NPs synergized with sorafenib inducing upregulation of cytochrome c and caspase-3 gene expression causing tumor cell death.

## 5. Conclusion

Finally, we conclude that ZnO-NPs synergized sorafenib to execute safer and effective antitumor activity leading to SEC growth reduction as shown in Figure 4 with a low cytotoxic effect on normal cells. Therefore, new therapeutic strategies for cancer treatment including ZnO-NPs combined with sorafenib could be developed. Moreover, long-term toxicity studies are required to rule out any long-term side effects regarding the combination.

## Figures and Tables

**Table 1:** Tumor growth inhibition rate and difference in tumor weights. Values are expressed as *M* ± SD of 10 animals in each group.

Parameters	SEC	MTX	ZnO-NPs	Sorafenib	ZnO-NPs+sorafenib	*P*
Tumor weight (g)	2.62 ± 0.20	1.22 ± 0.13^a^	1.41 ± 0.28^a^	1.32 ± 0.14^a^	1.07 ± 0.21^a^	<0.001^∗∗^
Tumor growth inhibition (%)	—	43.72	19.09^b^	35.17^c^	46.23^c^	<0.001^∗∗^

SD: standard deviation; *P*: probability; ^∗^significance < 0.05; ^∗∗^high significance. The test used one-way ANOVA followed by post hoc Tukey. ^a^Significant compared to the control SEC group. ^b^Significant compared to the MTX group. ^c^Significant compared to the ZnO-NP group. ^d^Significant compared to the sorafenib group.

**Table 2:** Hematological and biochemical parameters. Values are expressed as *M* ± SD of 10 animals in each group.

Parameters	Control	SEC	MTX	ZnO-NPs	Sorafenib	ZnO-NPs+sorafenib	*P*
Hematological parameters							
HB (g/L)	103.1 ± 7	78.3 ± 13.5^a^	94.6 ± 2.9	88 ± 13	83 ± 2.6	87.6 ± 8.1	<0.001^∗∗^
Leucocytes (cells × 10^3^/mm^3^)	5.1 ± 0.4	13.5 ± 0.9^a^	5.4 ± 0.58^b^	6.6 ± 2.72^b^	6.33 ± 1.5^b^	4.8 ± 0.83^b,d^	<0.001^∗∗^
RBCs (cells × 10^6^/mm^3^)	4.1 ± 0.3	3.13 ± 0.62	3.71 ± 0.15	3.66 ± 0.46	3.86 ± 0.61	3.53 ± 0.57	<0.001^∗∗^
Renal parameters							
Urea (mmol/L)	7.3 ± 0.41	11.2 ± 1.7^a^	5.8 ± 05^b^	9.9 ± 1.3^c^	8.9 ± 1.3	6.3 ± 0.9^b,d^	<0.001^∗∗^
Creatinine (mmol/L)	0.07 ± 0.002	0.21 ± 0.02^a^	0.09 ± 0.04^b^	0.09 ± 0.04^b^	0.12 ± 0.01^a,b^	0.08 ± 0.02^b,e^	<0.001^∗∗^
Hepatic parameters							
ALT (U/L)	45.6 ± 5.8	76.96 ± 9.1^a^	36.2 ± 4.05^b^	54.23 ± 12.8^b,c^	63.9 ± 12.62^a,c^	41.8 ± 14.4^b,e^	<0.001^∗∗^
AST (U/L)	64.8 ± 10.6	192.9 ± 8.3^a^	121 ± 7.76^a,b^	111.63 ± 17.1^a,b^	96.2 ± 7.96^a,b^	87.36 ± 4.37^a,b,c^	<0.001^∗∗^

SD: standard deviation; *P*: probability; ^∗^significance < 0.05; ^∗∗^high significance. The test used one-way ANOVA followed by post hoc Tukey. ^a^Significant compared to the control group. ^b^Significant compared to the control SEC group. ^c^Significant compared to the MTX group. ^d^Significant compared to the ZnO-NP group. ^e^Significant compared to the sorafenib group.

**Table 3:** Parameters of oxidative stress, cell viability assay, DNA fragmentation, and cytochrome c. Values are expressed as *M* ± SD of 10 animals in each group.

Parameters	SEC	MTX	ZnO-NPs	Sorafenib	ZnO-NPs+sorafenib	*P*
Tumor tissue						
MTT	0.49 ± 0.06	0.32 ± 0.05^a^	0.38 ± 0.08^a^	0.34 ± 0.05^a^	0.26 ± 0.10^a,c^	<0.001^∗∗^
DNA content (*μ*g/100 mg)	744.5 ± 62.9	421.9 ± 32.7^a^	532.5 ± 52.5^a^	621.5 ± 130.4^b^	365.6 ± 41.9^a,c,d^	<0.001^∗∗^
Cytochrome c (ng/mL)	0.32 ± 0.05	0.87 ± 0.15^a^	0.76 ± 0.10^a^	0.74 ± 0.14^a^	0.92 ± 0.18^a^	<0.001^∗∗^
ROS (FU/mg)	380 ± 60	730 ± 31^a^	530 ± 63^a,b^	650 ± 72^a^	790 ± 12^a,c^	<0.001^∗∗^
NO (nmol/100 mg tumor)	82.03 ± 16.6	66.23 ± 14.47	83.10 ± 14.90	72.76 ± 4.32	75.8 ± 15.01	<0.001^∗∗^
GSH (nmol/100 mg tumor)	328.4 ± 28.9	240.5 ± 30.6^a^	202.5 ± 72.3^a^	194.9 ± 33.1^a,b^	175.1 ± 31^a,b^	<0.001^∗∗^
MDA (nmol/100 mg tumor)	10.86 ± 1.20	18.73 ± 1.87^a^	19.3 ± 1.03^a^	14.26 ± 1.26^a^	22.40 ± 1.53^a,d^	<0.001^∗∗^
CAT (U/g tumor)	170.3 ± 11.2	97.4 ± 9.8^a^	84.6 ± 5.8^a^	110.5 ± 8.7^a^	77.9 ± 5.3^a,d^	<0.001^∗∗^
SOD (U/g tumor)	8.9 ± 1.5	4.2 ± 1.37^a^	3.9 ± 1.04^a^	5.4 ± 1.13^a^	3.2 ± 1.2^a,d^	<0.001^∗∗^
Serum TAC (*μ*mol/L)	0.5 ± 0.05	0.91 ± 0.05^a^	0.8 ± 0.14^a^	0.54 ± 0.07^a,b,c^	0.72 ± 0.08^a,b,d^	<0.001^∗∗^

SD: standard deviation; *P*: probability; ^∗^significance < 0.05; ^∗∗^high significance. The test used one-way ANOVA followed by post hoc Tukey. ^a^Significant compared to the control SEC group. ^b^Significant compared to the MTX group. ^c^Significant compared to the ZnO-NP group. ^d^Significant compared to the sorafenib group.

## Data Availability

Data used to support the findings of this study are available from the corresponding author upon request.
